# A design-based framework for optimal stratification using super-population models with application on real data set of breast cancer

**DOI:** 10.1371/journal.pone.0323619

**Published:** 2025-05-22

**Authors:** Faizan Danish

**Affiliations:** Department of Mathematics, School of Advanced Sciences, VIT-AP University, Inavolu, Beside AP Secretariat, Amaravati, Andhra Pradesh, India; Universidad Santiago de Cali, COLOMBIA

## Abstract

This study investigates the determination of stratification points for two study variables within the framework of simple random sampling, with a focus on estimating the population mean using a closely related auxiliary variable. Employing a superpopulation model, the research aims to minimize overall variance by deriving simplified equations that enhance the precision of parameter estimates. Instead of categorizing variables, the study emphasizes continuous variables to establish optimal strata boundaries (OSB), which are essential for creating homogeneous groups within each stratum. This stratification leads to more efficient sample sizes (SS) and improved accuracy in parameter estimation. However, achieving optimal OSB and SS poses challenges in scenarios with a fixed total sample size, such as survey designs constrained by limited budgets. To address this, the study proposes a robust methodology for calculating OSB and SS, leveraging knowledge of the survey’s per-unit stratum measurement costs or its probability density function. An empirical application of the method is demonstrated using breast cancer data, where the mean perimeter is estimated based on mean radius and mean texture. Additionally, hypothetical examples using Cauchy and standard power distributions are provided to illustrate the versatility of the proposed approach. The newly developed method has been integrated into the updated stratifyR package and implemented in LINGO software, facilitating its practical application. Comparative analysis reveals that this approach consistently outperforms or matches existing methods in enhancing the precision of population parameter estimation. Furthermore, simulation studies confirm its higher relative efficiency, making it a valuable contribution to the field of stratified sampling.

## 1. Introduction

The problem of optimal stratification was first explored in [1], laying the foundation for subsequent research. Building on this, [2] extended the investigation by considering variables as stratification factors under Neyman allocation, focusing on univariate cases as an extension of [1]. However, handling multiple features in estimation complicates achieving straightforward optimum allocation. Previous studies, such as [3,4], have examined proportional allocation techniques, particularly for cases involving two features. Numerous methods have been proposed across different contexts, as demonstrated by [4–9]. Addressing the multivariate stratification problem, [10] introduced an algorithm employing a penalized objective function optimized via the Simulated Annealing technique. Similarly, [11] proposed algorithms for stratifying asymmetric populations using power allocation to estimate sample sizes. In another notable approach, [12] utilized Dynamic Programming combined with Neyman allocation to address stratification issues, assuming a Weibull distribution for the stratification variable. Several R packages, such as GA4 Stratification (https://cran.r-project.org/src/contrib/Archive/GA4Stratification/), stratifyR ([13]), and sample (available on R CRAN), offer practical tools for stratification. To address inconsistencies between stratification and study variables, [14] introduced a method using two models. Over the decades, stratification methods have been broadly classified into approximation and optimization techniques [15]. Among notable developments, an exact resource allocation technique was presented in [16], employing algorithms such as BRKGA (Biased Random Key Genetic Algorithm) and GRASP (Greedy Randomized Adaptive Search Procedure) [17]. Additionally, [18] applied a dynamic programming approach to compute stratification points for two correlated variables. The study referenced stratification points derived through various methods, measuring accuracy using percentage relative efficiency based on variance estimates. Survey precision and implementation costs often require balancing, as decision-making is hindered by insufficient cost-related data. This study addresses these challenges by optimizing the variance function under basic cost constraints, formally linking survey precision with cost [19–21]. In cases of limited cost and variance information, approximate stratified designs based on cost considerations become necessary. Recent survey data, often the most representative, provides valuable insights for planning. Within total survey cost constraints, this technique minimizes population mean variance. Optimal stratification inherently involves allocating sample sizes effectively based on preconstructed strata [1,5]. The core principle lies in partitioning the population into Optimal Strata Boundaries (OSB) to minimize total stratum variance for a given sample size. The determination of OSB has been extensively studied, initially by Dalenius, who used the study variable as the primary stratification criterion [22–26]. Approximation methods, such as partitioning square root cumulative frequencies into equal intervals, have also been explored [27]. While the cumulative root frequency method remains popular, its arbitrary nature has been critiqued [28,29] presents a methodology for achieving approximately optimal stratification for sensitive quantitative variables in probability proportional to size (PPS) sampling. The proposed approach is demonstrated through theoretical formulation and empirical applications, improving the precision of estimates in stratified sampling designs. Alternatives, such as the Geometric method, were developed for skewed populations but lacked universal applicability [30–33]. In a recent development, [34] proposed a methodology for OSB and optimal sample size (OSS) determination, leveraging known per-unit measurement costs or the probability density function of the survey. They demonstrated this approach using Wave 18 of the HILDA Survey dataset to determine average annual disposable income in Australia, providing insights relevant to policy decisions. Further advancements in OSB determination include dynamic programming (DP) approaches, which divide populations into subsets to maximize precision [35,36]. Refinements by [37] explored precise OSB under Neyman allocation but excluded cost constraints, complicating practical implementation.

In this study, two study variables were employed as stratification variables for the target variable. Breast cancer patient data were analyzed to evaluate the precision of the proposed method.

## 2. Optimum strata boundaries and sample size allocation

Let L X M be strata of size $N_{hk}$, h = 1, 2,…,L, k = 1,2,…,M are formed from a population of size N, which is $N=\sum_{h}\sum_{k}N_{hk}$, he population mean for the study variable involves calculating the sum of the products of the number of units in each stratum and subgroup, then dividing by the total population size as:


$${\overset{―}{y}}_{st}=\frac{1}{N}\sum_{h=1}^{L}\sum_{k=1}^{M}N_{hk}{\overset{―}{y}}_{hk}$$

In case of SRSWOR having $n_{hk}$ points, the desired average of any point $(i,j{)}^{th}$ in $(h,k{)}^{th}$ stratum $\left(y_{hij}\right)$ can be calculated using the weighted mean as below:


$${\overset{―}{y}}_{hk}=\frac{1}{n_{hk}}\sum_{i=1}^{n_{h}}\sum_{j=1}^{n_{k}}y_{hij}$$

The Unbiased Estimator of the Population Mean in the case of Stratified Sampling is


$${\overset{―}{y}}_{st}=\sum_{h=1}^{L}\sum_{k=1}^{M}W_{hk}{\overset{―}{y}}_{hk}\;$$

and its Variance is


$$Var\left({\overset{―}{y}}_{st}\right)=\sum_{h}\sum_{k}\left(\frac{1}{n_{hk}}-\frac{1}{N_{hk}}\right)W_{hk}^{2}S_{hk}^{2}\;$$ (1)

in which $W_{hk}=\frac{N_{hk}}{N}$ denotes the weight of the $(h,k{)}^{th}$ stratum. While as


$$S_{hk}^{2}=\frac{1}{N_{hk}-1}\sum_{i=1}^{n_{h}}\sum_{j=1}^{n_{k}}{\left(y_{hij}-{\overset{―}{Y}}_{hk}\right)}^{2}$$

represents the Variance of the $(h,k{)}^{th}$ stratum and $n_{hk}$ denotes the sample size to be taken from the $(h,k{)}^{th}$ stratum. In order to estimate the variance of the sample for the estimation of true variance, the stratum variance is represented by the sample variance.

There are several allocation methods for allocating the sample size for each stratum. Such as Equal allocation, Proportional allocation, Neyman allocation, and Optimum allocation. The best choice of allocation method will lead to the better precision and accuracy. So, it is challenging stage to choose the best way to allocate sample size. The allocation method will also depend on the total sample size, the variance and the cost associated with the $(h,k{)}^{th}$ stratum. The cost involved for each unit makes the allocation method difficult, if the cost allocated for each unit is same the Neyman allocation minimizes the variance (1) best, however if the cost varies for each and every unit, the optimum allocation may be utilized for allocation of sample size.

Let us consider the Cost function as


$$C=C_{1}+\sum_{h=1}^{l}\sum_{k=1}^{n}C_{hk}n_{hk}$$ (2)

where $C_{1}\;$represents the overhead cost, encompassing expenses related to administration, conducting interviews, training sessions, and other associated activities, and $C_{hk}$is the mean per unit cost of $(h,k{)}^{th}$ stratum as


$$C_{hk}=\sum_{i=1}^{n_{h}}\sum_{j=1}^{n_{k}}C_{hkij}$$

where $C_{hkij}$ is cost associated to the $(i,j{)}^{th}$ unit in the $(h,k{)}^{th}$ stratum. It is to be noted here that the cost $C_{hkij}$ will differ from unit to unit. To estimate the unit cost the prior information may be utilized.

The average costs can be obtained as


$$C_{hk}=\frac{1}{W_{hk}}\int C\;f(c)\;dc$$

where f(c) is the Probability Density Function of unit cost measurement.

In order to determine the optimum sample size $n_{hk}$to be taken from $(h,k{)}^{th}$ stratum, we optimize the $V\left({\overset{―}{y}}_{st}\right)$ defined in (1) subject to the total cost involved defined in (2).

Using Lagrangian multiplication technique, we can estimate the sample size


$$n_{hk}=\frac{C-C_{1}}{\sum_{h}\sum_{k}W_{hk}S_{hk}\sqrt{C_{hk}}}\;\frac{W_{hk}S_{hk}}{\sqrt{C_{hk}}}$$ (3)

The Optimum Allocation method ensures that a larger sample size is selected within a stratum when there is greater heterogeneity and if the variance within the stratum is large.

While using (3) in (1), we have


$$V_{opt}\left({\overset{―}{y}}_{st}\right)=\frac{{\left(\sum_{h}\sum_{k}W_{hk}S_{hk}\sqrt{C_{hk}}\right)}^{2}}{C-C_{1}}-\frac{\sum_{h}\sum_{k}W_{hk}^{2}S_{hk}^{2}}{N_{hk}}$$ (4)

Since we cannot minimize the overhead cost and total sample size, so in order to minimize (4) we can minimize (5), if FPC is ignored.


$$\sum_{h}\sum_{k}W_{hk}S_{hk}\sqrt{C_{hk}}$$ (5)

Hence, the total variance to be minimized is given in equation (5).

In Sample Surveys we can have various targeted variables and it is not necessary that the information available on the study variable is sufficient for stratification. However, a help taken from a related variables who are easily available may lead to better precision. Hence, let $X_{1}$ and $X_{2}$ are two auxiliary variables associated with the study variable ‘Y’. These two auxiliary variables will be utilized as stratification variables. $X_{1}$ and $X_{2}$ have the continuous distribution with the Probability Density Function $f\left(x_{1}\right)$ and $f\left(x_{2}\right)$ respectively. $x_{1}\in (a,b)$ and $x_{2}\in (c,d)$. The total number of strata to be made L X M, where (a,c) and (b,d) are the first and last values of $X_{1}$ and $X_{2}$variables. Then the total range for $X_{1}$ and $X_{2}$ are


$$b-a=R_{1\;}and\;d-c=R_{2}$$ (6)

In nutshell we need to find the points between (a,b) and (c,d) so that variance defined in equation (5) is minimum.

Let’s consider the functional relationship between the study variable and the auxiliary variable be


$$y=f\left(x_{1},x_{2}\right)+e$$

where $f\left(x_{1},x_{2}\right)$ be the function of $X_{1}$ and $X_{2}$which may be linear or non-linear and ‘e’ is the error defined as


$$E\left(\frac{e}{X_{1}},X_{2}\right)=0,\;V\left(e/X_{1},X_{2}\right)=\eta >0$$

Let the intermediate points for $X_{1}$ and $X_{2}$ be


$$a={x_{1}}_{0}\leq {x_{1}}_{1}\leq {x_{1}}_{2}\leq {x_{1}}_{3}\leq \ldots \leq {x_{1}}_{L}=b$$

and


$$c={x_{2}}_{0}\leq {{{x_{2}}_{1}\leq x}_{2}}_{2}\leq {x_{2}}_{3}\leq \ldots \leq {x_{2}}_{M}=d$$

and we have to obtain these points except $x_{1_{0}},x_{1_{L}},x_{2_{0}}\;and\;x_{2_{M}}$ as they will be known and fixed points we need to estimate subject to the minimization of equation (5).

The Probability Density Function of $X_{1}$ and $X_{2}$ should be be known for stratification variables, the estimated $W_{hk\;}and\;S_{hk}^{2}$ in equation (5) can be obtained by


$$W_{hk}=\int_{x_{2_{k-1}}}^{x_{2_{k}}}\int_{x_{1_{h-1}}}^{x_{1_{h}}}f(x_{1},x_{2}dx_{1}\;dx_{2}$$ (7)

$$S_{hk}^{2}=\frac{1}{W_{hk}}\int_{x_{2_{k-1}}}^{x_{2_{k}}}\int_{x_{1_{h-1}}}^{x_{1_{h}}}{\left(x_{1}x_{2}\right)}^{2}f(x_{1},x_{2}dx_{1}\;dx_{2}-\mu_{hk}^{2}$$ (8)

where


$$\mu_{hk}=\frac{1}{W_{hk}}\int_{x_{2_{k-1}}}^{x_{2_{k}}}\int_{x_{1_{h-1}}}^{x_{1_{h}}}\left(x_{1}x_{2}\right)f(x_{1},x_{2}dx_{1}\;dx_{2}$$ (9)

and $\left(x_{1_{h-1}},\;x_{1_{h}}\right)$, $\left(x_{2_{k-1}},\;x_{2_{k}}\right)$ denotes the boundary points of $(h,k{)}^{th}$ stratum. Thus, it can be seen from equations (7)-(9) that all the terms can be expressed in terms of boundary points of $(h,k{)}^{th}$ stratum.

Thus equation (5) can be expressed as a function of boundary points $\left(x_{1_{h-1}},x_{1_{h}}\right)$ and $\left(x_{2_{k-1}},x_{2_{k}}\right)$ as


$$f\left(\;x_{1_{h-1}},\;x_{1_{h}}\;\right),\;\left(\;x_{2_{k-1}},\;x_{2_{k}}\;\right)=W_{hk}S_{hk}\sqrt{C_{hk}}$$

Such that the estimator of OSB can be calculated in finding the intermediate points ${x_{1}}_{1},{x_{1}}_{2},..,{x_{1}}_{L}$ and ${x_{2}}_{1},{x_{2}}_{2},\ldots ,{x_{2}}_{k}$ then the following problem

optimize $f\left[\left(\;x_{1_{h-1}},\;x_{1_{h}}\;\right),\;\left(\;x_{2_{k-1}},\;x_{2_{k}}\;\right)\right]=W_{hk}S_{hk}\sqrt{C_{hk}}$

subject to the constraints


$$a={x_{1}}_{0}\leq {x_{1}}_{1}\leq {x_{1}}_{2}\leq {x_{1}}_{3}\leq \ldots ..\leq {x_{1}}_{L}=b$$

$$c={x_{2}}_{0}\leq {{{x_{2}}_{1}\leq x}_{2}}_{2}\leq {x_{2}}_{3}\leq \ldots ..\leq {x_{2}}_{M}=d$$

with $R_{1_{h}}=(\;x_{1_{h}}-x_{1_{h-1}})$ and $R_{2_{k}}=(\;x_{2_{k}}\;-x_{2_{k-1}})$ denotes the total width of $(h,k{)}^{th}$ stratum. Thus, the total deviation can be expressed in equation (6) can be denoted in strata width as:


$$\sum_{h=1}^{L}R_{1_{h}}=\sum_{h=1}^{L}\left(\;x_{1_{h}}-x_{1_{h-1}}\right)=x_{1_{L}}-x_{1_{0}}=R_{1}$$

and


$$\sum_{k=1}^{M}R_{2_{k}}=\sum_{k=1}^{M}\left(\;x_{2_{k}}-x_{2_{k-1}}\right)=x_{2_{M}}-x_{2_{0}}=R_{2}$$

We can define the $(h,k{)}^{th}$ strata point as:


$$x_{1_{h}}=x_{1_{0}}+R_{1_{1}}+R_{1_{2}}+\ldots +R_{1_{h}}=x_{1_{h-1}}+R_{1_{h}}$$

$$x_{2_{k}}=x_{2_{0}}+R_{2_{1}}+R_{2_{2}}+\ldots +R_{2_{k}}=x_{2_{k-1}}+R_{2_{k}}.$$

The issue of estimating the Optimum Sample Size (OSB) can be approached as a quest for determining the Optimum Strata Width (OSW), represented by the sum of widths within strata $R_{1_{1}}+R_{1_{2}}+\ldots +R_{1_{L}}$ and $R_{2_{1}}+R_{2_{2}}+\ldots +R_{2_{k}}$. This can be formulated as the following mathematical programming problem:

Optimize


$$\sum_{h}\sum_{k}f_{hk}(R_{1_{h}},x_{1_{h-1}},{R_{2_{k}},x}_{2_{k-1}})$$

Subject to the constraints


$$\begin{array}{c} \sum_{h}R_{1_{h}}=R_{1}\\ \sum_{k}R_{2_{k}}=R_{2} \end{array}$$ (10)

and $R_{1_{h}}\geq 0,\;R_{2_{k}}\geq 0\;$,

 h=1,2,…L and k=1,2,…M.

With the given initial value $x_{1_{0}}$, the first term of the objective function of (10) $f_{11}(R_{1_{1}},{x_{1}}_{1-1},\;R_{2_{1}},{x_{2}}_{1-1})$ will be a function of $R_{1_{1}}$ and $R_{2_{1}}$ only. Once $R_{1_{1}}$ and $R_{1_{2}}$ is determined initially, then the next term ill be the function of $R_{1_{2}}$ and $R_{2_{2}}$ only and the same pattern will continue.

Due to the exceptional pattern of the MPP (10) we can solve it by using dynamic programming approach. Hence the MPP (10) can be written as the function of $R_{1_{h}}$ and $R_{2_{k}}$ only as follows

optimize $\sum_{h}\sum_{k}f_{hk}(R_{1_{h}},R_{2_{k}})$

Subject to the constraints


$$\begin{array}{c} \sum_{h}R_{1_{h}}=R_{1}\\ \sum_{k}R_{2_{k}}=R_{2} \end{array}$$ (11)

and $R_{1_{h}}\geq 0,\;R_{2_{k}}\geq 0\;$,

 h=1,2,…L and k=1,2,…M.

## 3. Solution procedure using dynamic programming

It can be observed that the problem defined in (11) is a problem of many stages in which the main objective function and the constants are sums of separable function of $R_{1_{h}}$ and $R_{2_{k}}$. Considering the nature of the problem, dynamic programming emerges as a viable solution approach. Dynamic programming entails resolving optimization problems by decomposing them into more manageable subproblems and storing their solutions in a table for later retrieval. This method involves solving each subproblem just once and retaining its solution, thus eliminating the need for redundant computations. By efficiently combining the solutions to the overall problem to be found. This technique is widely applied in various fields such as algorithm design, artificial intelligence, economics and bioinformatics, providing an efficient approach to tackle complex problem into optimal solutions as given in (11).

Now let us assume a fraction of problem defined in (11) for $\left(l_{1},m_{1}\right)<(L,M)$

Optimize $\sum_{h=1}^{l_{1}}\sum_{k=1}^{m_{1}}f_{hk}\left(R_{1_{h}},R_{2_{k}}\right)$

Subject to the constraints


$$\sum_{h=1}^{l_{1}}R_{1_{h}}=r_{1_{h}}$$

$$\sum_{k=1}^{m_{1}}R_{2_{k}}=r_{2_{k}}$$

$$R_{1_{h}}\geq 0,\;R_{2_{k}}\geq 0,\;$$

 h=1,2,…L and k=1,2,…M.

where $r_{1_{h}}<R_{1}$ and $r_{2_{k}}<R_{2}$ the total deviation to be subdivided into $\left(l_{1},m_{1}\right)$ strata. It is to be noted here that

$r_{1_{h}}=R_{1}$ and $r_{2_{k}}=R_{2}$ if $L_{1}=L$ and $M_{1}=M$

and


$$r_{1_{h}}=R_{1_{1}}+R_{1_{2}}+\ldots +R_{1_{h}}$$

$$r_{2_{k}}=R_{2_{1}}+R_{2_{2}}+\ldots +R_{2_{k}}$$

which are the transformation function as:


$$\begin{array}{c} r_{1_{h}}=R_{1_{1}}+R_{1_{2}}+\ldots +R_{1_{h}}\\ r_{1_{h-1}}=R_{1_{1}}+R_{1_{2}}+\ldots +R_{1_{h-1}}=r_{1_{h}}-R_{1_{h}}\\ \begin{array}{c} r_{1_{h-2}}=R_{1_{1}}+R_{1_{2}}+\ldots +R_{1_{h-2}}=r_{1_{h-1}}-R_{1_{h-1}}\\ \begin{array}{c} \vdots \\ \begin{array}{c} r_{1_{2}}=R_{1_{1}}+R_{1_{2}}=r_{1_{3}}-R_{1_{3}}\\ r_{1_{1}}=R_{1_{1}}=r_{1_{2}}-R_{1_{2}} \end{array} \end{array} \end{array} \end{array}$$

Similarly, we have


$$\begin{array}{c} r_{2_{k}}=R_{2_{1}}+R_{2_{2}}+\ldots +R_{2_{k}}\\ r_{2_{k-1}}=R_{2_{1}}+R_{2_{2}}+\ldots +R_{2_{k-1}}=r_{2_{k}}-R_{2_{k}}\\ \begin{array}{c} \vdots \\ r_{2_{2}}=R_{2_{1}}+R_{2_{2}}=r_{3}-R_{2_{3}}\\ r_{1}=R_{2_{1}}=r_{2}-R_{2_{2}} \end{array} \end{array}$$

Let $\psi_{l_{1}Xm_{1}}(R_{1_{1}},R_{2_{1}})$ denotes minimum value of the function (21), that is


$$\psi_{l_{1}Xm_{1}}\left(r_{1_{1}},r_{2_{1}}\right)=Min\left[\sum_{h=1}^{l_{1}}\sum_{k=1}^{m_{1}}\psi_{hk}\left(r_{1_{h}},r_{2_{k}}\right)/\sum_{h=1}^{l_{1}-1}R_{1_{h}}=r_{1_{h-1}},\sum_{k=1}^{m_{1}-1}R_{2_{k}}=r_{2_{k-1}}\right]$$

and $R_{1_{h}}\geq 0,\;R_{2_{k}}\geq 0,\;h=1,2,\ldots \;l_{1},\;k=1,2,\ldots m_{1}$

with the above definition of $\psi_{l_{1}Xm_{1}}\left(R_{1_{1}},R_{2_{1}}\right)$, the MPP defined in equation (11) is equation to finding $\psi_{LXM}(R_{1},R_{2})$ recursively by defining $\psi_{l_{1}Xm_{1}}(R_{1_{1}},R_{2_{1}})$ for $l_{1}=1,2,\ldots ,L$ and $m_{1}=1,2,\ldots .,M$,


$$0\leq r_{1_{1}}\leq R_{1},\;0\leq r_{2_{1}}\leq R_{2}.\;$$

$$\psi_{l_{1}Xm_{1}}\left(r_{1_{1}},r_{2_{1}}\right)=Min\left[\psi_{l_{1}Xm_{1}}\left(R_{1_{1}},R_{2_{1}}\right)+\frac{\sum_{h=1}^{l_{1}-1}\sum_{k=1}^{m_{1}-1}\psi_{hk}\left(R_{1_{h}},R_{2_{k}}\right)}{\sum_{h=1}^{l_{1}-1}R_{1_{h}}=r_{1_{h}}-R_{1_{h}},\sum_{k=1}^{m_{1}-1}R_{2_{k}}=r_{2_{k}}-R_{2_{k}}}\right]$$

and $R_{1_{h}}\geq 0,\;R_{2_{k}}\geq 0,\;h=1,2,\ldots \;l_{1},\;k=1,2,\ldots m_{1}$

For the fixed value of $\left(R_{1_{1}},R_{1_{2}}\right)$, $0\leq r_{1_{1}}\leq R_{1_{1}},\;0\leq r_{2_{1}}\leq R_{2_{1}}$.


$$\psi_{l_{1}Xm_{1}}\left(r_{1_{1}},r_{2_{1}}\right)=\varphi_{l_{1}Xm_{1}}\left(R_{1_{1}},R_{2_{1}}\right)+Min\left[\sum_{h=1}^{l_{1}-1}\sum_{k=1}^{m_{1}-1}\psi_{hk}\left(R_{1_{1}},R_{2_{k}}\right)/\sum_{h=1}^{l_{1}-1}R_{1_{h}}=r_{1_{h}}-R_{1_{h}},\sum_{k=1}^{m_{1}-1}R_{2_{k}}=r_{2_{k}}-R_{2_{k}}\right]$$

$$R_{1_{h}}\geq 0,\;R_{2_{k}}\geq 0,\;h=1,2,\ldots \;l_{1},\;k=1,2,\ldots m_{1}$$

$$1\leq l_{1}\leq L,1\leq m_{1}\leq M.$$

Utilizing the same methodology, we can derive the forward recursive equation for the dynamic programming problem, ultimately leading us to determine the optimal strata boundaries.

Let’s consider the functional relationship between the study variable and auxiliary variable to be linear, as depicted below:


$$y=a_{1}+a_{2}x_{1}+a_{3}x_{2}+e$$

then


$$\sigma_{hky}^{2}=a_{2}^{2}\sigma_{hkx_{1}}^{2}+a_{3}^{2}\sigma_{hkx_{2}}^{2}$$

where

$W_{hk},\sigma_{hkx_{1}}^{2}$ and $\sigma_{hkx_{2}}^{2}$denotes the weights and variances of $x_{1}$ and $x_{2}$ and are given by


$$W_{hk}=\int_{x_{1_{h-1}}}^{x_{1_{h}}}\int_{x_{2_{k-1}}}^{x_{2_{k}}}f(x_{1},x_{2}dx_{1}\;dx_{2}$$ (12)

$$\sigma_{hkx_{1}}^{2}=\frac{1}{W_{hk}}\int_{x_{2_{k-1}}}^{x_{2_{k}}}\int_{x_{1_{h-1}}}^{x_{1_{h}}}x_{1}^{2}f(x_{1}dx_{1}\;dx_{2}-\mu_{hkx_{1}}^{2}$$ (13)

$$\sigma_{hkx_{2}}^{2}=\frac{1}{W_{hk}}\int_{x_{1_{h-1}}}^{x_{1_{h}}}\int_{x_{2_{k-1}}}^{x_{2_{k}}}x_{2}^{2}f(x_{2}dx_{2}\;dx_{1}-\mu_{hkx_{2}}^{2}$$ (14)

where


$$\mu_{hkx_{1}}=\frac{1}{W_{hk}}\int_{x_{2_{k-1}}}^{x_{2_{k}}}\int_{x_{1_{h-1}}}^{x_{1_{h}}}x_{1}f\left(x_{1}\right)\;dx_{1}\;dx_{2}$$

$$\mu_{hkx_{2}}=\frac{1}{W_{hk}}\int_{x_{1_{h-1}}}^{x_{1_{h}}}\int_{x_{2_{k-1}}}^{x_{2_{k}}}x_{2}f\left(x_{2}\right)\;dx_{2}\;dx_{1}$$

## 4. Numerical illustration

**Fig 1:**
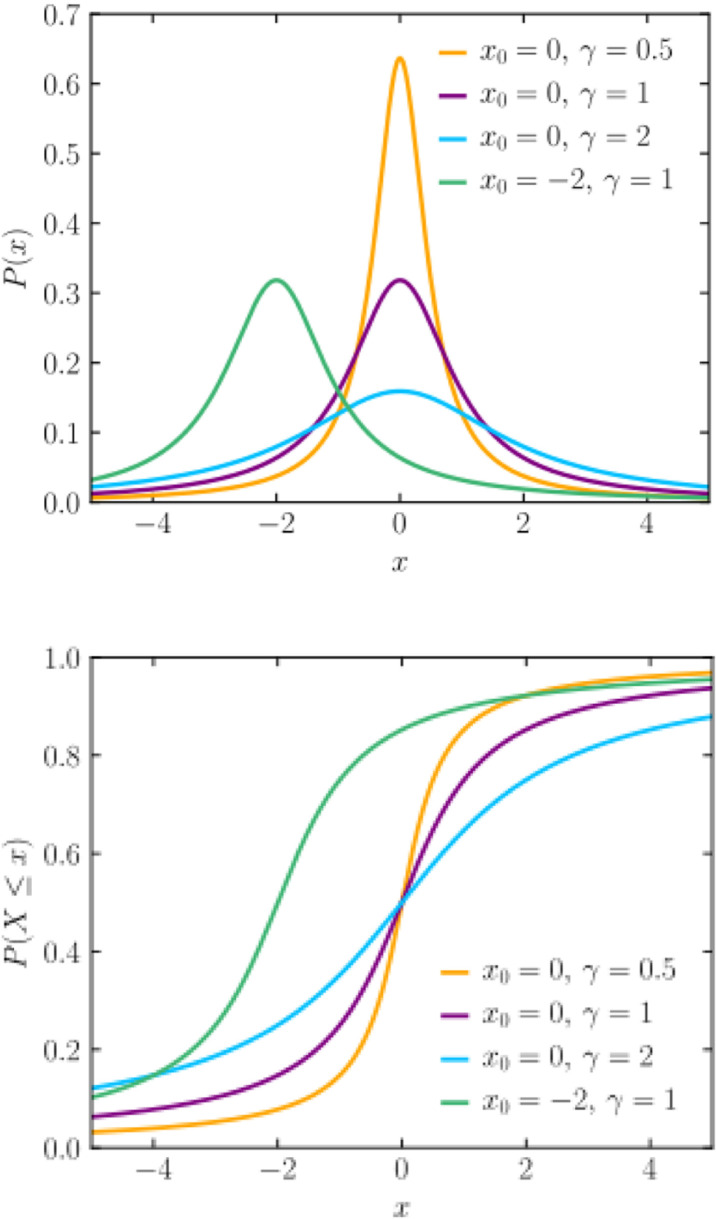
PDF and CDF of Cauchy distribution. These graphs illustrate the characteristic shape and behaviour of the Cauchy distribution, showcasing its heavy-tailed nature and symmetry around the mean.

To illustrate the computation details of the proper design, we can assume certain distributions of the auxiliary variables. The Cauchy distribution, named in honor of the mathematician Augustin-Louis Cauchy, is a probability distribution commonly encountered in statistics and physics. It finds significant applications in various domains, notably in fields like optics and signal processing. It is characterized by its heavy tails, meaning that it has a higher probability of extreme outcomes compared to other distributions with finite variance. The Cauchy distribution lacks a well-defined mean or variance due to its heavy tails, making it a challenge to work with in some statistical contexts. Despite its mathematical quirks, the Cauchy distribution finds applications in modelling phenomena with uncertain or heavy-tailed behaviour, such as financial markets and turbulent fluid dynamics. As for graphs, since the Cauchy distribution has heavy tails, plotting its probability density function often reveals a distribution that peaks at the centre and rapidly decays towards the tails, resembling a symmetric “bell” shape. Some of the examples of a probability density function (PDF) and a cumulative distribution function (CDF) graph of the Cauchy distribution which is presented in Fig 1 as below:

Let $X_{1}\;$follow a distribution named after Augustin Cauchy, which is continuous with a probability density function as provided below.


$$f\left(x_{1}\right)=\frac{1}{\pi t\left[1+{\left(\frac{x_{1}-\theta^{\prime }}{t}\right)}^{2}\right]};\ldots \ldots -\infty <x_{1}<\infty$$

where $x^{\prime }$ represents the location parameter, indicating the peak location of the distribution, and ‘t’ denotes the scale parameter, representing the half-width at half maximum, the standard Cauchy Distribution emerges as a special case. When θ=0 and t=1, it’s referred to as the standard Cauchy Distribution. Its probability density function is expressed as follows:


$$f\left(x_{1}\right)=\{\frac{1}{\pi \left(1+x_{1}^{2}\right)},\;-\infty <x_{1}<\infty \;$$

**Fig 2:**
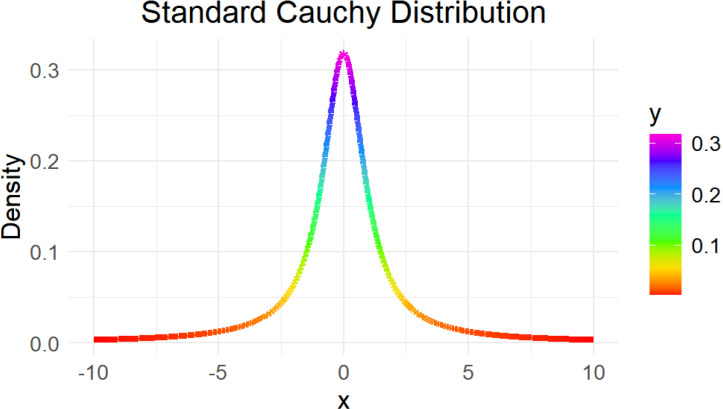
Distribution of ith standard cauchy.

The standard power distribution is a statistical model frequently utilized across diverse disciplines due to its versatility in capturing a wide array of phenomena. This distribution is characterized by two parameters: δ, which determines its shape, and θ, which sets its scale. The probability density function (PDF) of the standard power distribution is given by a formula that encapsulates the interplay between these parameters. Additionally, important statistical properties such as moments (mean, variance, etc.) are associated with this distribution, providing insights into its behavior and characteristics. The standard power distribution is applied in reliability engineering to model the lifetimes of components and systems. By analyzing failure rates and predicting reliability, engineers can optimize maintenance schedules and improve product performance. Survival analysis, prevalent in medical studies and epidemiology, investigates the time until an event (e.g., death) occurs. The standard power distribution proves useful in modeling survival times, aiding in understanding disease progression and treatment effectiveness. In queuing systems, this distribution can represent service times or interarrival times of customers. Queuing theory utilizes these models to optimize service levels, reduce waiting times, and enhance operational efficiency. The standard power distribution finds applications in modeling financial phenomena such as stock price movements and trading volumes. By understanding the distribution of duration between events, analysts gain insights into market dynamics and risk assessment. Environmental studies utilize the standard power distribution to analyze the timing of natural events like floods or species extinctions. By modeling these durations, researchers can assess environmental risks and develop strategies for mitigation and adaptation Fig 2 presents the distribution of standard power distribution of Cauchy distribution as below:

let us assume that the other auxiliary variable $X_{2}$ follows standard power distribution as


$$f\left(x_{2}\right)=\left\{ \begin{array}{c} \frac{\delta {x_{2}}^{\delta -1}}{\theta^{\delta }},\;x_{2}\geq \theta \;\\ 0,\;otherwise\; \end{array}\right.\;$$

using the equations (12) to (14), the values of $W_{hk},\;\mu_{hk}$ and $\sigma_{hk}^{2}$ can be computed


$$W_{hk}=\frac{\left[P_{1}\right]\left[P_{2}\right]}{\pi \theta^{\delta }}$$ (15)

$$\sigma_{hkx_{1}}^{2}=\frac{R_{2_{k}}}{4\pi \theta^{\delta }{\left(P_{1}P_{2}\right)}^{2}}4\left({R_{1_{h}}-P}_{1}\right){\left(P_{1}P_{2}\right)}^{3}-\pi R_{2_{k}}^{2}{\left\{\theta^{\delta }\log\left[\frac{1+{\left(R_{1_{h}}+x_{1_{h-1}}\right)}^{2}}{1+x_{1_{h-1}}^{2}}\right]\right\}}^{2}$$ (16)

$$\sigma_{hkx_{1}}^{2}=\frac{\pi \delta }{{\left(P_{1}P_{2}\right)}^{2}}\left\{R_{1_{h}}\left(P_{1}P_{2}\mathrm{\backslash rightleft}[P_{3}\right]-\pi \delta \left[P_{4}\right]\right\}\;$$ (17)

where


$$P_{1}=\tan^{-1}\left(R_{1_{h}}+x_{1_{h-1}}\right)-\tan^{-1}\left(x_{1_{h-1}}\right)$$

$$P_{2}=\left[{\left(R_{1_{k}}+x_{2_{k-1}}\right)}^{\delta }-{\left(x_{2_{k-1}}\right)}^{\delta }\right]$$

$$P_{3}=\left[{\left(R_{2_{k}}+x_{2_{k-1}}\right)}^{\delta +2}-{\left(x_{2_{k-1}}\right)}^{\delta +2}\right]$$

and


$$P_{4}=\left[{\left(R_{2_{k}}+x_{2_{k-1}}\right)}^{\delta +1}-{\left(x_{2_{k-1}}\right)}^{\delta +1}\right]$$

Substitute the values obtained in equations (15) to (17) in the optimization, we get

Optimize


$$\sum_{h}\sum_{k}\frac{\left[P_{1}\right]\left[P_{2}\right]}{\pi \theta^{\delta }}\sqrt{\begin{array}{c} \frac{a_{2}^{2}R_{2_{k}}}{4\pi \theta^{\delta }{\left(P_{1}P_{2}\right)}^{2}}4{\left(R_{1_{h}}-P_{1}\mathrm{\backslash rightleft}(P_{1}P_{2}\right)}^{3}-\pi R_{2_{k}}^{2}{\left\{\theta^{\delta }\log\left[\frac{1+{\left(R_{1_{h}}+x_{1_{h-1}}\right)}^{2}}{1+x_{1_{h-1}}^{2}}\right]\right\}}^{2}\\ +\frac{a_{3}^{2}\pi \delta }{{\left(P_{1}P_{2}\right)}^{2}}\left\{R_{1_{h}}\left(P_{1}P_{2}\right)\left[P_{3}\right]-\pi \delta \left[P_{4}\right]\right\}C_{hk} \end{array}}$$ (18)

Subject to the constraints


$$\sum_{h}R_{1_{h}}={r_{1}}_{h}$$

$$\sum_{k}R_{2_{k}}={r_{2}}_{k}$$

$\forall \;R_{1_{h}}\geq 0,R_{2_{k}}\geq 0,\;h=1,2,\ldots L$ and k=1,2,…M.

In order to the OSB we need to define the auxiliary variables in a finite interval such as defining $X_{1}$ in the interval , i.e., ${r_{1}}_{h}=1$ and the other variable $X_{2}$ with the same interval [0,1]. Further, it is assumed that the value of δ=3 and θ=1. Then the optimization problem defined in equation (18) can be written as


$$\sum_{h}\sum_{k}\frac{\left[P_{1}\right]R_{2_{k}}}{\pi }\sqrt{\begin{array}{c} \frac{a_{2}^{2}R_{2_{k}}}{4\pi \theta^{\delta }{\left(P_{1}P_{2}\right)}^{2}}4\left(R_{1_{h}}-P_{1}\right){\left(P_{1}R_{2_{k}}\right)}^{3}-\pi R_{2_{k}}^{2}{\left\{\log\left[\frac{1+{\left(R_{1_{h}}+x_{1_{h-1}}\right)}^{2}}{1+x_{1_{h-1}}^{2}}\right]\right\}}^{2}\\ +\frac{a_{3}^{2}3\pi }{{\left(P_{1}R_{2_{k}}\right)}^{2}}\left\{R_{1_{h}}R_{2_{k}}\left(P_{1}\right)\left[P_{3}^{1}\right]-3\pi \left[P_{4}^{1}\right]\right\}C_{hk} \end{array}}$$

where


$$P_{3}^{1}={\left(R_{2_{k}}+x_{2_{k-1}}\right)}^{5}-{\left(x_{2_{k-1}}\right)}^{5}$$

$$P_{4}^{1}={\left(R_{2_{k}}+x_{2_{k-1}}\right)}^{4}-{\left(x_{2_{k-1}}\right)}^{4}$$

subject to the constraints


$$\sum_{h}R_{1_{h}}=1$$

$$\sum_{k}R_{2_{k}}=1$$

$\forall \;R_{1_{h}}\geq 0,R_{2_{k}}\geq 0,\;h=1,2,\ldots L$ and k=1,2,…M.

Using the above optimization problem the solution was obtained using LINGO and the optimum strata boundaries are obtained presented in Tables 1 and 2 below for L X M (2 X 3). The cost of the units has been assumed to be 1,2,3,…,6 respectively $C_{11}=1,C_{12}=2,C_{13}=3,\;C_{21}=4,C_{22}=5,C_{23}=6$.

**Table 1:** Layout of OSB.

1.0000		
0.7948		
0.2794		
0.0000	0.5621	1.0000

**Table 2:** OSB and total variance.

OSB ${(x}_{1_{h}}.x_{2_{k}})$	TOTAL VARIANCE
(0.5621, 0.2794)	0.000127
(1.0000, 0.2794)	
(0.5621, 0.7948)	
(1.0000,0.7948)	
(1.0000,1.0000)	

## 5. An application on read data set of breast cancer

**Fig 3:**
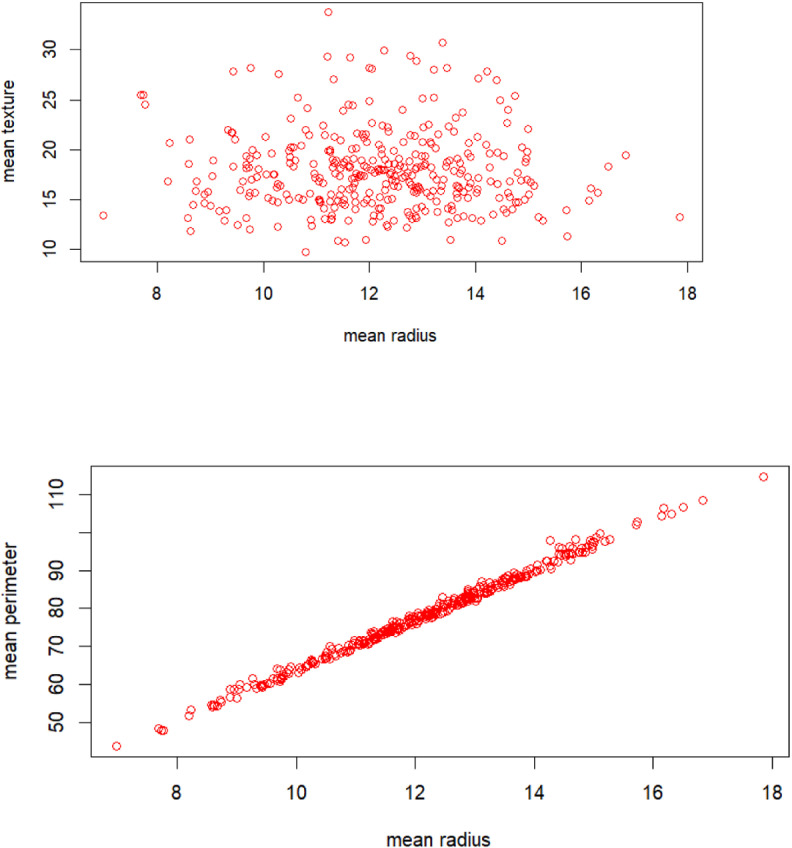
Distribution of mean radius with mean texture and mean perimeter.

**Fig 4:**
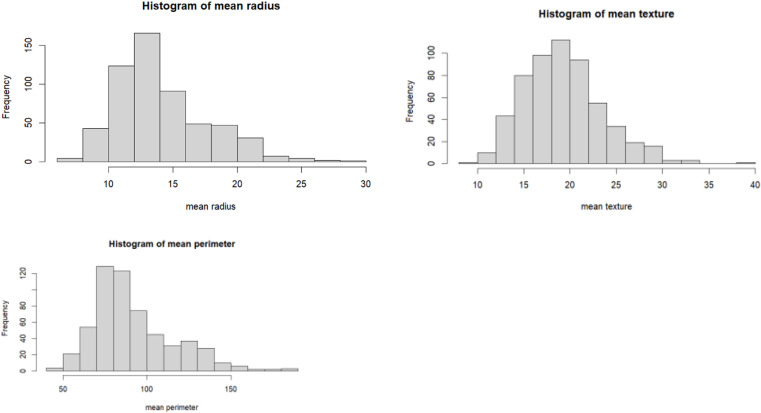
Distribution of the data in histogram.

**Fig 5:**
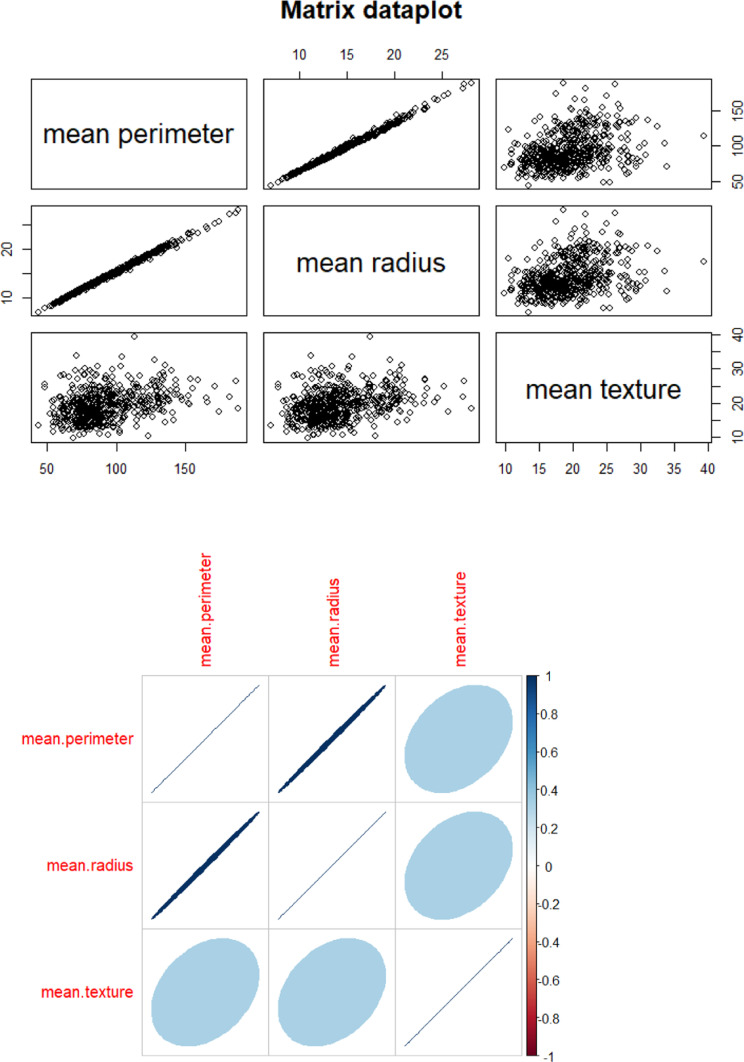
Correlation matrix plot.

Breast cancer represents a significant health concern globally, particularly among women. It is characterized by the abnormal growth of cells in breast tissue, often leading to the formation of tumours. With diverse subtypes and varying degrees of aggressiveness, breast cancer poses complex challenges in diagnosis, treatment, and management. Early detection through screening methods such as mammography plays a crucial role in improving prognosis and survival rates. Additionally, advancements in treatment modalities, including surgery, chemotherapy, radiation therapy, and targeted therapies, have contributed to improved outcomes for many patients. Despite these advancements, breast cancer remains a leading cause of cancer-related mortality worldwide, highlighting the ongoing need for research, education, and advocacy efforts to enhance prevention, early detection, and treatment strategies. I am incorporating breast cancer data in my this as a pivotal component of the study. This is a data set from UC repository [38]. This is part of my self-learning journey, tried different ML algorithms over this data set. The data set has 31 predictor variables and one target variable. The target variable has 2 class, whether the tumor is cancerous and non-cancerous. The inclusion of this data allows for a comprehensive analysis of various factors related to breast cancer, including risk factors, diagnostic procedures, treatment outcomes, and survival rates. By leveraging breast cancer data, my study aims to contribute to a deeper understanding of the disease and its impact on affected individuals and communities. Furthermore, utilizing this dataset enables the exploration of novel approaches to early detection, personalized treatment strategies, and interventions aimed at improving patient outcomes and quality of life. Overall, the incorporation of breast cancer data serves as a cornerstone in advancing knowledge and informing evidence-based practices in the field of oncology and public health. In this dataset we are using mean perimeter as a study variable and mean radius and mean texture as the auxiliary variables. The presentation of the data are displayed in Figs 3–5.

While fitting the model the estimated coefficients obtained are:


$$\begin{array}{c} \text{Mean\textbackslash{} perimeter }=\;-5.7899+6.8643\;*\text{ mean\textbackslash{} radius }+0.0406\;*\text{ mean\textbackslash{} texture} \end{array}$$ (19)

**Fig 6:**
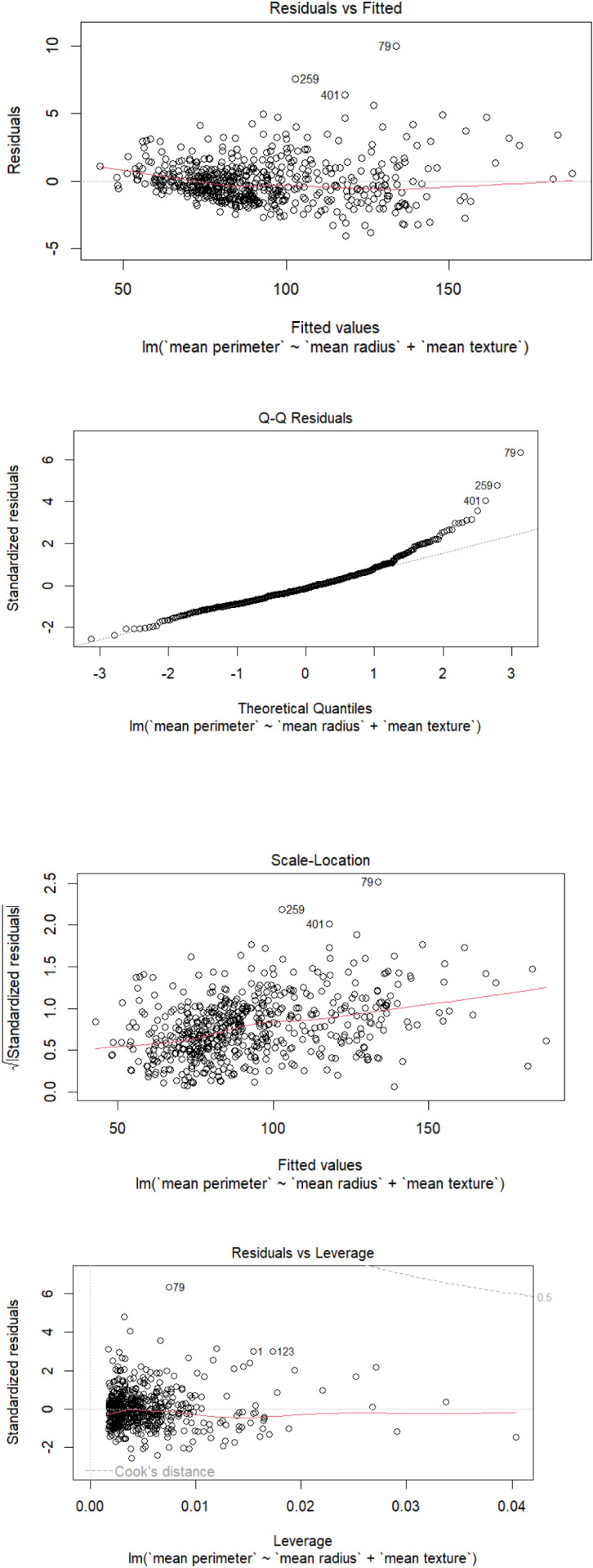
Presentation of the model.

The Fig 6 demonstrates the behaviour of the model.

Keeping under consideration the model obtained in equation (19) and substitute the equation (18), we obtained the strata boundaries along with the sample size of total 569 assuming the cost of units same for a total of 20 (5 × 4) strata presented in Table 3 along with total variance.

**Table 3:** OSB, Sample Size and total variance.

OSB $(x_{1_{h}},x_{2_{k}})$	Sample Size $\left(n_{hk}\right)$	Total variance
(11.42, 15.49)	30	590.4404795
(11.42, 21.62)	32	
(11.42, 32.79)	35	
(11.42, 39.28)	26	
(17.95, 15.49)	30	
(17.95, 21.62)	29	
(17.95, 32.79)	28	
(17.95, 39.28)	23	
(21.43, 15.49)	37	
(21.43, 21.62)	22	
(21.43, 32.79)	25	
(21.43, 39.28)	26	
(25.73, 15.49)	28	
(25.73, 21.62)	19	
(25.73, 32.79)	23	
(25.73, 39.28)	24	
(28.11, 15.49)	26	
(28.11, 21.62)	33	
(28.11, 32.79)	35	
(28.11, 39.28)	38	

**Fig 7:**
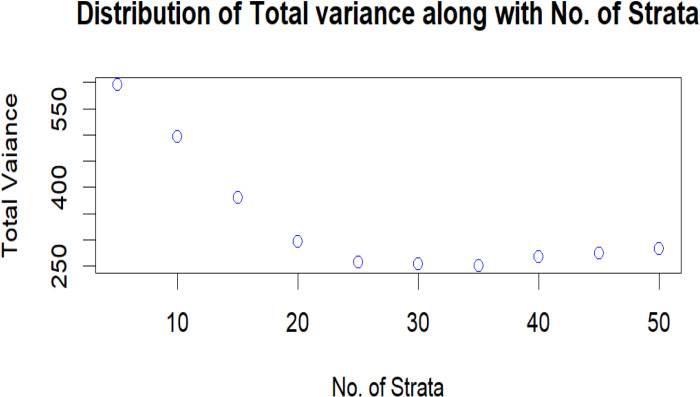
Variance distribution and number of strata.

Fig 7 demonstrates the variance corresponding to the number of strata and it can be observed the variance shows decreasing trend but up to the 25 number of strata only from which it shows increasing trend again. Hence it can be assessed from this graph that the number of strata should be taken under consideration while obtaining the Optimum strata boundaries.

## 6. Simulation study

To validate the effectiveness of the proposed stratification method, a simulation study was conducted. The primary objective was to assess its precision and compare its performance against several established methods. This evaluation involved the application of dynamic programming and was carried out using the R statistical software. The comparative methods included in this study are as follows:

1. **Dalenius and Hodges (1959)** cumulative (cum) method [39]: A widely recognized method for stratification that divides cumulative frequencies into equal intervals.
2. **Gunning and Horgan (2004)** geometric method [40]: Known for its suitability in skewed populations by incorporating geometric intervals for stratification.
3. **Lavallee-Hidiroglou (1988)** method [41]: A stratification approach refined by Kozak (2004) [42]: to improve applicability to asymmetric data distributions.
4. **Khan et al. (2018)** mathematical programming approach [43]:: A contemporary optimization-based method for determining stratification boundaries.
5. **Proposed method**: A novel stratification method introduced in this study, leveraging dynamic programming to minimize total variance under cost constraints.

### Simulation design and implementation

A synthetic dataset of **10,000 observations** was generated for this study. The auxiliary variable used in the simulation followed a **uniform distribution**, a standard choice for stratification studies due to its simplicity and controlled variance properties. The parameters for the uniform distribution were specified as a = 0.002 and b = 1.92.

Key statistics derived from the generated data include:

- Minimum value (a): 0.00395
- Maximum value (b): 1.8962
- Total deviation (k): k = b − a = 1.89225

Using this dataset, stratification points were calculated for the proposed method and the comparative methods mentioned above. The primary evaluation criterion was total variance, a critical measure for determining stratification effectiveness.

### Stratification points and variance

The stratification points identified by the proposed method were compared to those obtained using the other methods. Variances were calculated for each method under varying numbers of strata (L) to assess their relative performance. The results are summarized in Table 4 below:

**Table 4:** Total variance obtained by different methods.

L (Number of Strata)	Cumulative Method	Geometric Method	Lavallee-Hidiroglou Method	Khan et al. (2018)	Proposed Method
**2**	0.9878	1.0243	1.2556	0.7931	0.6212
**3**	0.8358	0.8717	0.7926	0.4305	0.3444
**4**	0.6857	0.8092	0.7584	0.3656	0.2846
**5**	0.5888	0.7152	0.6926	0.2687	0.2652
**6**	0.5197	0.6448	0.6847	0.2305	0.2208

### Results and analysis

From the Table 4 the Detailed Observations are:

1. **Performance Across Strata Configurations**:
2. The proposed method consistently achieved the lowest total variance for all configurations of LL, ranging from L = 2 to L = 6. For L = 2, the variance achieved by the proposed method (0.6212) was significantly lower than that of the cumulative method (0.9878) and the geometric method (1.0243), highlighting its efficiency even with fewer strata.
3. **Comparison with Traditional Methods**:
4. The cumulative method, while widely used, exhibited higher variances, particularly as L increased. The geometric method performed better than the cumulative method but still lagged behind the proposed approach.
5. **Advanced Techniques**
6. The Lavallee-Hidiroglou method, known for its robust application to asymmetric data, displayed variances higher than the proposed method, especially for L = 2 and L = 3. The Khan et al. (2018) mathematical programming approach, though more precise than traditional methods, was consistently outperformed by the proposed method.
7. **Impact of Increased Strata**:
8. Increasing the number of strata (L) generally reduced variance across all methods. However, the rate of improvement was most pronounced for the proposed method, demonstrating its adaptability to complex stratification scenarios.

**Advantages of the Proposed Method:**

- **Efficiency**: The proposed method achieves superior precision with lower variances across all tested scenarios.
- **Scalability**: Its performance remains consistent even as the number of strata increases, making it suitable for both simple and complex datasets.
- **Cost-Effectiveness**: By minimizing variance under cost constraints, the proposed method ensures practical applicability in survey design and resource allocation.

The simulation study highlights the effectiveness of the proposed method in achieving optimal stratification. By consistently outperforming traditional and advanced methods, the proposed approach demonstrates its potential for real-world applications requiring precise and cost-effective stratification. Its robust performance, coupled with the flexibility to handle varying strata configurations, makes it a valuable contribution to the field of stratified sampling and survey design.

## 7. Conclusion

This study explores a novel approach to optimal stratification for Cauchy and standard power distributions, focusing on determining stratification points and improving estimation precision. By leveraging closely related auxiliary variables, the proposed method enhances the accuracy of population estimates. It also facilitates Optimum Sample Allocation by considering the frequency distribution of auxiliary variables.Additionally, the study introduces a framework for determining Optimum Sample Size (OSB) and Sample Size (SS) within a stratified sampling design, accounting for budget constraints and varying per-unit measurement costs across strata. The stratification problem is formulated as a Mathematical Programming Problem, where stratum-wise costs are incorporated, and solutions are obtained using Dynamic Programming. The effectiveness of this approach is demonstrated through its application to breast cancer data, with similar promising results observed in simulations using Cauchy and standard power distributions. Comparative analysis shows that the proposed method performs as well as or better than conventional stratification techniques, making it a suitable choice for cost-sensitive survey planning. Unlike traditional methods, this research integrates financial constraints directly into OSB and SS estimation, ensuring practical applicability in real-world survey designs. Future research directions include developing advanced sampling strategies to further minimize data collection costs, optimizing sample sizes by balancing trade-offs between cost, mode, and statistical power, and exploring adaptive survey designs for cost efficiency. Additionally, Bayesian methods, leveraging prior information, could enhance the precision of sample allocation. The incorporation of neutrosophic statistics in complex data environments also presents a promising avenue, offering a flexible alternative to classical statistical approaches, offers a potential avenue for future research, building on existing studies in this area [44] and [45].
